# Supplementary material for: HIV-1 derived oligonucleotides induce a type I IFN/STING dependent immune suppression reversible by targeting IFNARI
Source: PLoS Pathog. 2026 Jan 13;22(1):e1013868. doi: 10.1371/journal.ppat.1013868 (PMC12826489; doi:10.1371/journal.ppat.1013868)
Supplement: S2 Table — (DOCX) [file ppat.1013868.s002.docx]

|  | **Target** | **Clone** | **Fluorochrome** | **Working dilution** | **Company** |
| --- | --- | --- | --- | --- | --- |
| **Panel 1** |  |  |  |  |  |
|  | CD3 | SK7 | BV750 | 160 | BD Biosciences |
|  | CD4 | Sk3 | cFlour V450 | 50 | Cytek Biosciences |
|  | CD8 | SK1 | cFlour V547 | 100 | Cytek Biosciences |
|  | CD28 | L293 | BV480 | 200 | BD Biosciences |
|  | CD69 | FN50 | APC-H7 | 50 | BD Biosciences |
|  | HLADR | L243 | BV570 | 40 | BioLegend |
|  | CD38 | HIT2 | R720 | 50 | Cytek Biosciences |
|  | PD1 | J116 | PE-Cy5.5 | 40 | Novus Biologicals |
|  | CTLA4 | BNI3 | BB515 | 50 | BD Biosciences |
| **Panel 2** |  |  |  |  |  |
|  | CD1c | F10/21A3 | BV421 | 50 | BioLegend |
|  | PDL1 | MIH1 | BB515 | 50 | BD Biosciences |
|  | CD86 | 2331 (FUN-1 | BV650 | 100 | BD Biosciences |
|  | Viability | - | Via Red Dye | 1000 | Cytek Biosciences |
