# Supplementary figures and images for: HIV-1 derived oligonucleotides induce a type I IFN/STING dependent immune suppression reversible by targeting IFNARI
Source: PLoS Pathog. 2026 Jan 13;22(1):e1013868. doi: 10.1371/journal.ppat.1013868 (PMC12826489; doi:10.1371/journal.ppat.1013868)

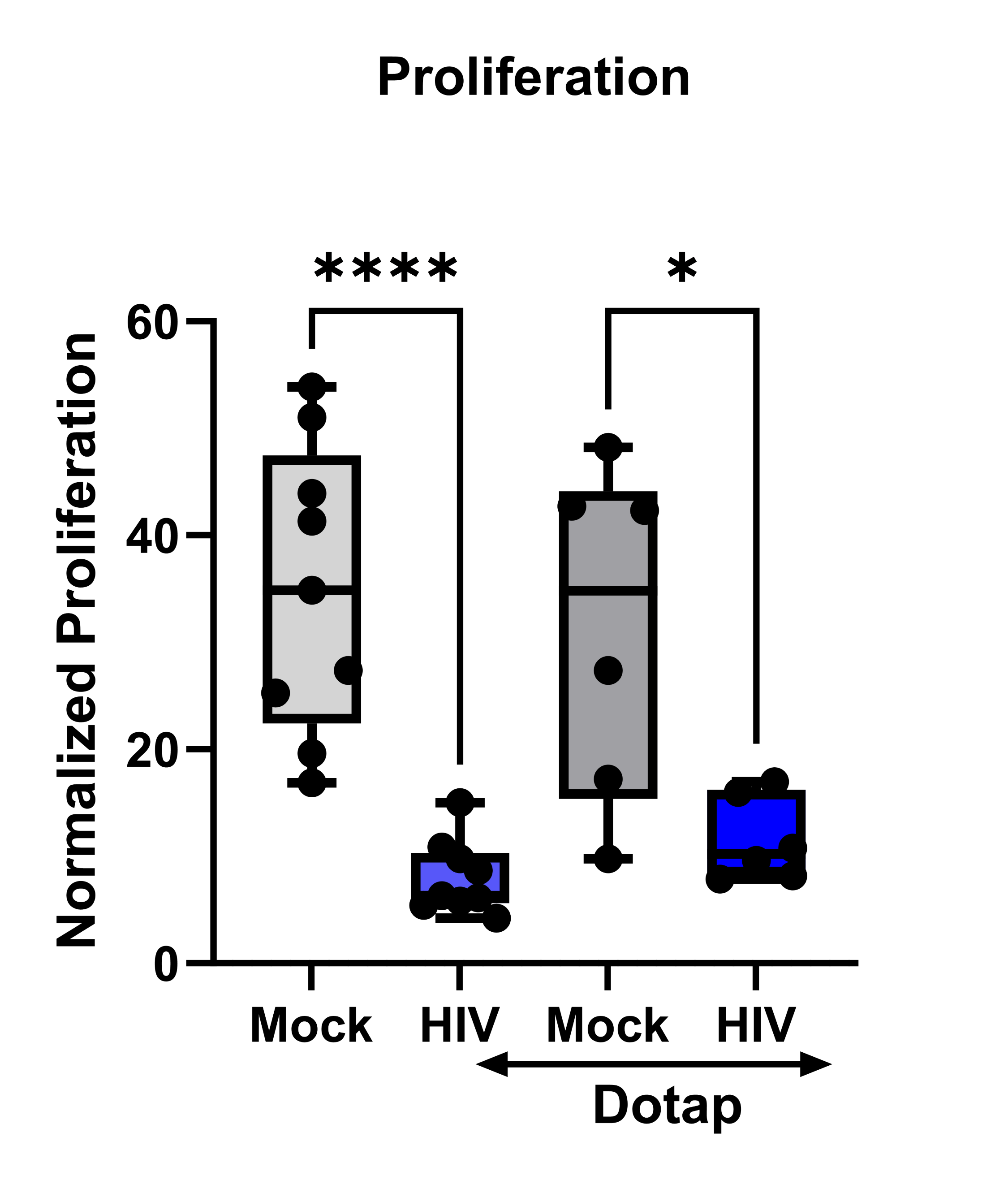

Supplement: S1 Fig — Mature DCs exposed to mock or HIV-1 BaL were untreated or treated by DOTAP transfection. The different DC groups were cocultured with naïve T cells (1:10) and the DC-T cell coculture was restimulated on day 7 with the same DCs as the initial stimulation. One day after restimulation, the T proliferation was assessed via 3H thymidine incorporation. Statistical significance was determined using the ANOVA. * = p-value <0.05, ** = p-value <0.01, *** = p-value <0.001, **** = p-value <0.0001. (TIF) [file ppat.1013868.s003.tif]

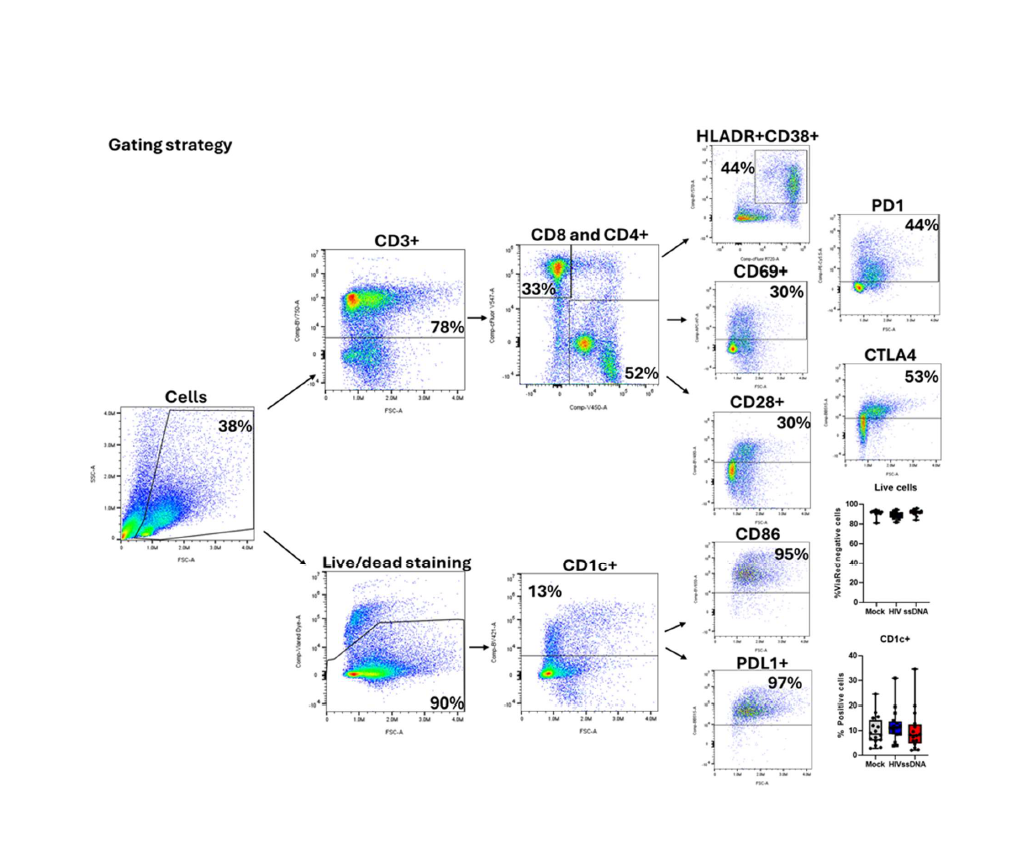

Supplement: S2 Fig — Mature DCs exposed to mock, HIV-1 or DOTAP transfected with ssDNA were cocultured with naïve T cells (in a ratio of 1:10) and the DC-T cell coculture was restimulated on day 7 with the same DCs as the initial stimulation. After 16-18h of incubation, on day 8, cells were harvested and stained for spectral flow cytometry. (TIF) [file ppat.1013868.s004.tif]
